# Why *ONE* Is More Than 5

**DOI:** 10.1371/journal.pbio.1001235

**Published:** 2011-12-20

**Authors:** Catriona J. MacCallum

**Affiliations:** Public Library of Science, Cambridge, United Kingdom

*PLoS ONE* is five years old this month. Though still young in age, the journal has grown up remarkably rapidly, to the extent that it is now the largest peer-reviewed journal in the world. In the past five years, it has both garnered huge respect and support from authors, readers, and editors, and drawn the criticism and ire of many commercial publishers and establishment figures still fighting to maintain the science publishing status quo. Their fight now appears to be in vain, however: this past year a series of journals emerged that are very similar in scope to *PLoS ONE* (Table 1), suggesting that the landscape of scholarly publishing has irreversibly shifted. *PLoS ONE* clearly fills an unmet need in the world of scientific publishing, or publishers and scholarly societies wouldn't want to copy it.

**Table 1:** A sample of recently launched journals similar in scope to *PLoS ONE*.

Journal Name	Publisher	Website
G3	Genetics Society of America	http://www.g3journal.org
BMJ Open	British Medical Journals publishing group	http://bmjopen.bmj.com
Scientific Reports	Nature Publishing Group	http://www.nature.com/srep
AIP Advances	American Institute of Physics	http://aipadvances.aip.org/
Biology Open	Company of Biologists	http://bio.biologists.org/
TheScientificWorldJournal (TSWJ)	Hindawi	http://www.tswj.com/
QScience Connect	Bloomsbury Qatar Foundation	http://www.qscience.com/
SAGE Open	SAGE	http://sgo.sagepub.com/
Springer Plus	Springer	http://www.springeropen.com/springerplus/

Journals are included if they don't filter articles for publication based on perceived importance or interest.

The success of *PLoS ONE* has surprised even us. The journal is now publishing about 70 papers a day (i.e., currently around 4,000 papers every quarter), and this figure continues to grow (Figure 1). If the trend continues, it will publish 14,500 articles in 2011: approximately 1 in 60 of all the papers indexed by PubMed in that calendar year will have been published in *PLoS ONE*. It has even attracted a new term—“megajournal”—to characterize it and the other journals of its ilk [1].

**Figure 1:**
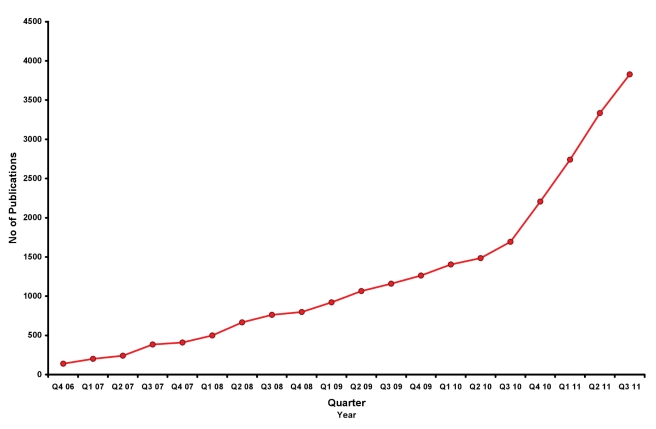
Publication growth of *PLoS ONE*. This is provided as the number of publications each quarter year since the last quarter of 2006, when *PLoS ONE* was launched.

We believe its success relies on two features: trust and innovation. By demonstrating that open access (OA) is compatible with high quality and rigorous science, *PLoS Biology*, then *PLoS Medicine* and the PLoS “Community Journals” (*PLoS Genetics*, *PLoS Computational Biology*, *PLoS Pathogens*, and *PLoS Neglected Tropical Diseases*) built a “PLoS brand,” making PLoS a trusted source of excellent science that authors and readers respect. As a result, PLoS could introduce a single key innovation beyond that of OA—one that represented a fundamental change to the traditional editorial model (and which has garnered awards from the Association of Learned and Professional Publishers [2] and the Scholarly Publishing and Academic Resources Coalition [3]). All articles in *PLoS ONE* are peer reviewed, but editors and reviewers are explicitly asked not to assess the “broad interest” or importance of a paper, a criterion that provides the rationale for other journals to reject articles. Instead, any article can be published if it is technically sound, ethically and appropriately reported, and its conclusions are supported by the data. Thus, *PLoS ONE* publications include negative results, methods papers, and studies that replicate (but do not duplicate) others, as well as articles that potentially represent a major advance for the field. And because *PLoS ONE* covers all of science (albeit with a current focus on the life and medical sciences), and because the publication fee ensures that each article covers its own editorial and production costs, there is no limit to *PLoS ONE*'s potential size beyond that of science itself [4].

## Growing Up: Responsibilities and Challenges

With size, however, come responsibilities and challenges [4]. A fundamental responsibility is to ensure that peer review is appropriately rigorous, regardless of subject area. Training and managing a growing editorial board of more than 2,500 academic editors from very diverse fields and cultures to oversee this process represents one of the ongoing challenges for *PLoS ONE*. This challenge is especially acute if these editors have been trained, by their prior activities in more traditional journals, to feel that part of their role is to increase a journal's impact factor by rejecting papers that they feel are not sufficiently novel or exciting (something that *PLoS ONE* explicitly does not attempt to do). Another responsibility is to ensure that basic standards of reporting are high. For example, each article must have appropriate ethical approval for the work carried out (be it on humans or animals), and authors and editors are asked to declare any financial or other competing interests (which are then made transparent both to the reviewers and to readers on any published article). Indeed, by publishing so many papers, *PLoS ONE* has an opportunity to help set reporting standards in science rather than follow existing ones. (Some countries and institutions, for example, have no independent ethical committee overseeing animal studies; although assessed on a case-by-case basis, such papers are generally rejected.) Every article submitted to *PLoS ONE*, therefore, goes through a series of rigorous checks to ensure that appropriate standards have been met, before an academic editor or reviewer even sets eyes on the paper.

## The Post-Publication Dawn?

The greatest challenge now, not just for *PLoS ONE* but for all OA publications, is not what happens to the article before it is published but what happens once it reaches the public domain. It seems almost bizarre to think that publishers traditionally felt their job finished once the paper was published and archived. With changing technology and social media, publication is now just the beginning of an article's “life.” This is where new opportunities to serve and advance science lie and why it is so important to ensure papers—and the data associated with them—are OA, i.e., not just free to read but free to reuse [5].

*PLoS ONE* and the other megajournals are giant OA content generators. But although *PLoS ONE* publishes more than 1,300 papers a month, it doesn't yet organize this massive content beyond allowing navigation by (author selected) subject categories. And although searching for articles is made easier by using PLoS's faceted search [6] or resources such as PubMed [7], we know that the audience comprises not just research scientists, but policy makers, health officials, educators, journalists, and the curious reader, all of whom will have different needs in terms of how they navigate and discover content. Moreover, no reader is interested in the content of only one journal, no matter how big; they want to reuse information regardless of its source. The question now is whether the content can not only be structured to cater to different communities, but also satisfy the needs of each individual and even enable them to generate new questions or discover novel avenues of research.

A very straightforward way to organize content is to package relevant articles into subject-specific collections. PLoS has a range of collections covering papers from all their journals (e.g., in *PLoS ONE* [8]), some of which are the outcome of specific conferences or projects (such as the Census of Marine Life [9]). Another solution is to provide “hubs” of activity around certain topics. One such initiative, still in the early stages of development, is the PLoS Biodiversity Hub [10], funded by the Sloan Foundation, which allows individuals from the community (curators) to select and filter articles, regardless of the venue, and which can be enhanced by comments from curators and via semantic linking.

It is likely, however, that the solution will not be to provide only pre-packaged platforms of content for readers to come and browse but to give individuals the tools to create their own personalized “hub” and to use, reuse, and track research in any direction they wish. There is a growing list of innovative research aids, such as Mendeley [11], which already enables you to organize your reference library and PDFs and to find others with similar interests; publishers would then just need to incorporate these as part of an article-level service. To help with this, PLoS and Mendeley recently launched a “binary battle” to build software applications (apps) that make science more open and useful for the reader using the application programming interface (API) provided by PLoS and/or Mendeley [12]. The possibilities, therefore, are limited only by imagination, and the next few years are likely to provide an exciting period of experimentation with different navigation tools.

## Alternative Thinking

In such a post-publication world, where greater emphasis is put on the article and its content rather than on the journal in which it happened to be published, how can you identify which papers and resources are more important for your work than others? Relying on the journal's Impact Factor to tell you about the merits of an individual paper is inappropriate [13],[14]. *PLoS ONE*, for example, has some papers that have received more than 200 citations, while 20% of articles one year or older have received more than nine citations and 76% one or more, a pattern of variation that will be familiar to all journals. By studying the citations to the individual articles, rather than a journal-level metric, a reader can understand in a much more nuanced way the research impact of that article. But impact also takes many different forms, some unmeasurable [14]. An article published in 2008 by *PLoS Medicine*, for example, provided a “Dirty War Index” [15], which has been adapted for use in NATO military environments, such as Southern Afghanistan, to reduce the possibility of injuring Afghan civilians. The approach has led to NATO changing procedures [16]. How can citations or downloads ever reflect this impact?

PLoS therefore provides a range of metrics [17] with each article in all the PLoS journals (which can be accessed via the “Metrics” tab on the online version). These include traditional parameters, such as the number of times an article is downloaded and how often it is cited (as recorded by different databases such as the Web of Science [18] and Scopus [19]) but also whether it has been bookmarked (e.g., by CiteULike [20]). The data about downloads and citations are also available under our OA license for anyone to download and analyze.

We have purposely provided an array of metrics because there is no one metric that can yet capture the different value readers place on articles. Moreover, this field is moving fast. An obvious addition might include how often and how soon an article is mentioned on Twitter or Facebook. But others are coming up with more innovative ideas. Several of the top “10+1” apps, shortlisted in the PLoS/Mendeley binary battle [12], for example, are about measuring the impact of articles or researchers.

## The Open-Access Ecosystem

The transition to sophisticated navigation and data extraction tools and alternative metrics will enhance all open-access articles to the benefit of science and scientists. Of more immediate concern, however, is that open-access publications still represent only about 8% of scientific publications [21]. This is going to change, not least because the new swath of recently launched OA megajournals will dramatically increase the market share. Such competition is good for OA and good for *PLoS ONE*, as it will promote innovation in the services that OA publishers provide to authors and readers, and help ensure that users get the best value from these services.

*PLoS ONE* turning 5 is an important milestone, but what is much more significant is the effects of *PLoS ONE* (and other successful OA journals) on the entire OA ecosystem and the future of publishing [22],[23]. And *PLoS ONE* wouldn't have achieved this without the pioneering authors who first published in *PLoS Biology* and the other PLoS journals, and the authors and editors who volunteered for the *PLoS ONE* experiment. Happy Birthday to all those who made and continue to make *PLoS ONE* happen.
